# Fibrillar Type I Collagen Enhances the Differentiation and Proliferation of Myofibroblasts by Lowering *α*2*β*1 Integrin Expression in Cardiac Fibrosis

**DOI:** 10.1155/2017/1790808

**Published:** 2017-01-30

**Authors:** Jian Hong, Ming Chu, Lijun Qian, Junhong Wang, Yan Guo, Di Xu

**Affiliations:** Department of Cardiology, Geriatric Medicine, The First Affiliated Hospital of Nanjing Medical University, Nanjing, China

## Abstract

Many studies have shown that *α*2*β*1 integrin plays an important role in the development of cardiac fibrosis. However, the mechanism of how *α*2*β*1 integrin regulates the differentiation and proliferation of myofibroblasts in cardiac fibrosis through fibrillar collagen (FC) remains uncertain. We established that FC mimicked the 3-dimensional extracellular matrix (ECM) of fibroblasts from post-myocardial infarction (MI) patients in vivo. This allowed us to explore the differentiation and proliferation of cardiac fibroblasts on FC. Here, we report that low expression of *α*2*β*1 integrin increased protein kinase B (AKT) activation and *α*-smooth muscle actin (*α*-SMA) expression. This occurred due to the instability of phosphatase and tensin homolog (PTEN) in myofibroblasts on FC. We also demonstrated that FC reduced protein phosphatase type 2A (PP2A) activity of myofibroblasts, which was coincident with low *α*2*β*1 integrin expression and activation of AKT, but not mitogen-activated protein kinase (ERK). In addition, knock-down of both *β*1 integrin and PP2A in fibroblasts promoted differentiation and proliferation via AKT activation and increased *α*-SMA expression. In summary, our study demonstrated that low *α*2*β*1 integrin expression regulated its downstream targets PTEN and AKT via crosstalk with PP2A, a critical cell signaling pathway that permits aberrant differentiation and proliferation of myofibroblasts on FC.

## 1. Introduction

Cardiac fibrosis is a common complication of various diseases, including post-myocardial infarctions (MI), and it can eventually lead to heart failure [1, 2]. Myofibroblasts are key contributors to fibrogenesis, a process characterized by the expression of *α*-smooth muscle actin (*α*-SMA) and production of collagen [3, 4]. However, the underlying mechanisms of regulating the differentiation of fibroblasts to myofibroblasts in cardiac fibrosis remain to be elucidated.

Fibrillar type I collagen plays a pivotal role as a potential inhibitor in the proliferation of normal fibroblasts in the extracellular matrix (ECM) [5, 6]. Under pathological condition of ECM remodeling in fibrosis, the accumulating fibrillar collagen (FC) can promote the differentiation and proliferation of myofibroblasts [7, 8]. Integrins are cell surface receptors, which hold important roles at the apex of cell signaling pathways modulating cell differentiation and proliferation [3, 5, 6]. *α*2*β*1 integrin is a major receptor of fibrillar type I collagen. Many studies have found that altering *β*1 integrin expression may result in increased myocardial dysfunction in MI and cardiac fibrosis [3, 9–11]. Importantly, integrin plays a crucial role in inducing the differentiation of cardiac fibroblasts to myofibroblast by cell signaling and mechanical stress of ECM remodeling as well [9, 12].

Protein phosphatase type 2A (PP2A) is a Ser/Thr phosphatase and has an important role in the control of cell growth and division [13]. Prior studies found that *α*2*β*1 integrin is required for PP2A activation in response to collagen matrices, and phosphatase and tensin homolog (PTEN) and phosphatidylinositol-4,5-bisphosphate 3-kinase (PI3K) play key roles in the regulation of myofibroblasts' function [6, 14–16]. Notably, low expression of *α*2*β*1 integrin or PTEN has been linked to fibroblast proliferation in lung fibrosis [6, 17]. These findings are consistent with our previous study that PTEN expression in cardiac fibroblasts was decreased in cardiac fibrosis after post-MI in mice [15]. However, the interaction of *α*2*β*1 integrin with FC in cardiac fibrosis remains unexplored. In this paper, we will address whether *α*2*β*1 integrin can lead to the differentiation and proliferation of myofibroblasts and identify the downstream targets of *α*2*β*1 integrin in myofibroblasts in cardiac fibrosis.

This is the first report indicating the interaction of *α*2*β*1 integrin with FC matrices is involved in the differentiation and proliferation of myofibroblasts in cardiac fibrosis. The abnormal expression of *α*2*β*1 integrin is associated with altered activity of PTEN/PP2A signaling, leading to increased AKT activity and *α*-SMA expression.

## 2. Materials and Methods

### 2.1. Isolation and Culture of Cardiac Fibroblasts

Cardiac fibroblasts were isolated from 3-month-old B6 mice as previously described [18]. Briefly, the mouse heart tissues were removed and minced with a razor blade. After digestion of the heart tissue with 0.4% type 2 collagenase (Thermo Fisher Scientific, Waltham, MA, USA), cells were pelleted and seeded in 10 cm polystyrene dishes and incubated for 120 minutes in the DMEM medium containing high glucose (Thermo Fisher Scientific, Waltham, MA, USA) with 10% fetal calf serum at 37°C with 5% CO_2_. After two hours, the medium was removed to eliminate cardiomyocytes, which did not attach to the plates, and replaced with fresh medium. Fibroblasts were allowed to grow to 70% confluence and then expanded at a ratio of 1 : 3. The experiments were performed only on cells at passage 3.

### 2.2. FC

The FC solution (final concentration, 0.75 mg/mL) was prepared with 1.25 mL type I collagen solution (3.1 mg/mL, Advanced BioMatrix, San Diego, CA, USA) and 0.9 mL 5x DMEM (pH 8.2) and diluted to 5 mL with 1x DMEM with 1% FBS. FC were formed following incubation of the solution at 37°C for 90 minutes [6].

### 2.3. Antibodies and Reagents

Anti-*β*1 integrin antibody was purchased from BD Biosciences (San Jose, CA, USA). Anti-PTEN, AKT, p-AKT Ser473, ERK, and p-ERK antibodies were purchased from Cell Signaling (Danvers, MA, USA). Anti-*α*2 integrin, GAPDH, and PP2A subunit C (PP2Ac) antibodies were obtained from Millipore (Millipore, Temecula, CA, USA). Anti-*α*-SMA antibody, wortmannin, and U0126 were purchased from Sigma-Aldrich (St Louis, MO, USA).

### 2.4. Cell Line

GD25 was *α*_2_*β*_1_ integrin-null fibroblast, and GD25 *α*_2_*β*_1_ integrin cells were GD25 cells reconstituted with *α*_2_*β*_1_ integrin in fibroblasts. Briefly, both types of cell were maintained in DMEM containing L-glutamine, sodium pyruvate, and 4.5 g/mL glucose and supplemented with 10% FBS and 1% penicillin and streptomycin at 37°C with 5% CO_2_. GD25 *α*_2_*β*_1_ integrin cells were kept under selection pressure with the addition of 10 *μ*g/mL puromycin [6, 19].

### 2.5. Cell Migration Assay

Cell migration was performed by Electric Cell-substrate Impedance Sensing (ECIS) (Applied BioPhysics, Troy, NY, USA), which was an impedance based method to study cell activities in tissue culture in real-time. We modified the protocol described by the manufacturer: 20,000 cells/cm2 were seeded in ECIS arrays (8 wells) and grew to about 100% confluent up to about 24 hrs. The cells were then killed by a small active electrode. The additional FC was polymerized in each well at 0.75 mg/mL for 90 minutes. The migration was assessed by continuing impendent measurements at 20 hours.

### 2.6. shRNA

*β*1 integrin, PTEN, PP2Ac, and control scramble shRNA obtained from Open Biosystems (Rockford, IL, USA) were incorporated into the pGIPZ lentiviral vector. The cells were infected at a multiplicity of infection (MOI) of 1 : 20.

### 2.7. Pulse-Chase Assay

The cells were washed twice with methionine-free DMEM medium and incubated for 45 minutes in methionine-free DMEM with 10% FBS. Cells were then incubated in methionine-free DMEM with 10% FBS containing ^35^S-methionine (300 *μ*Ci/mL) (NEN Life Science Products, Boston, MA, USA). After 45 minutes, the medium was replaced with complete medium. The cells were harvested and the cell lysate was resolved on 8% polyacrylamide gel. The PTEN labeled with ^35^S-methionine was quantified by phosphorimaging and normalized to the amount of beta-actin present as loading control by Western blot [20].

### 2.8. PP2A Phosphatase Activity Assay

PP2A activity was assessed by dephosphorylation of the phosphopeptide K-R-pT-I-R-R according to the manufacturer's instructions (Millipore, Temecula, CA, USA). Briefly, PP2Ac was immunoprecipitated from cell lysates with 4 *μ*g of anti-PP2Ac antibody, incubated with protein A agarose, and then washed with TBS. Immunoprecipitated PP2A was then incubated with 20 *μ*L Ser/Thr Assay Buffer and 60 *μ*L of the phosphopeptide substrate. The enzyme reaction was terminated by adding 100 *μ*L of Malachite Green Phosphate Detection Solution. PP2A activity was measured using a microtiter plate reader at 650 nm.

### 2.9. Semiquantitative and Real-Time PCR

RNA from mouse cardiac fibroblasts was isolated with TRI reagent (Sigma-Aldrich, St Louis, MO, USA) following the manufacturer's instructions. cDNA was synthesized from 1 *μ*g of total RNA using iScript cDNA Synthesis kit (Bio-Rad, Hercules, CA, USA). RT-PCR analysis was performed on an iCycler system (Bio-Rad). The following primers were used (5′-3′): *β*_1_ integrin: forward TTCAGACTTCCGCATTGGCTTTGG; reverse: TGGGCTGGTGCAGTTTTGTTCAC; *α*_2_ integrin, forward: TGTCACGATTCCCCTCATGA; reverse: TGCAGTCATAGCCAACAGCAA; beta-actin, forward: CGCCACCAGTTCGCCATGGA; reverse: TACAGCCCGGGGAGCATCGT; PTEN, forward: AATTCCCAGTCAGAGGCGCTATGT; reverse: GATTGCAAGTTCCGCCACTGAA CA [21, 22].

### 2.10. Proliferation Assay

Cells were seeded in DMEM medium with 10% FBS on day 1. On day 4, cells were harvested by trypsin digestion and counted.

### 2.11. Data Analysis

All experiments were repeated a minimum of three times. Data were expressed as mean ± SD. Data from Western blots were measured by densitometry with BioSpectrum Imaging System (UVP, Upland, CA, USA). All results were analyzed using a two-sided, unpaired *t*-test. A *p* value of <0.05 was deemed significant.

## 3. Results

### 3.1. FC Promoted the Differentiation and Proliferation of Cardiac Fibroblasts

**Figure 1:**
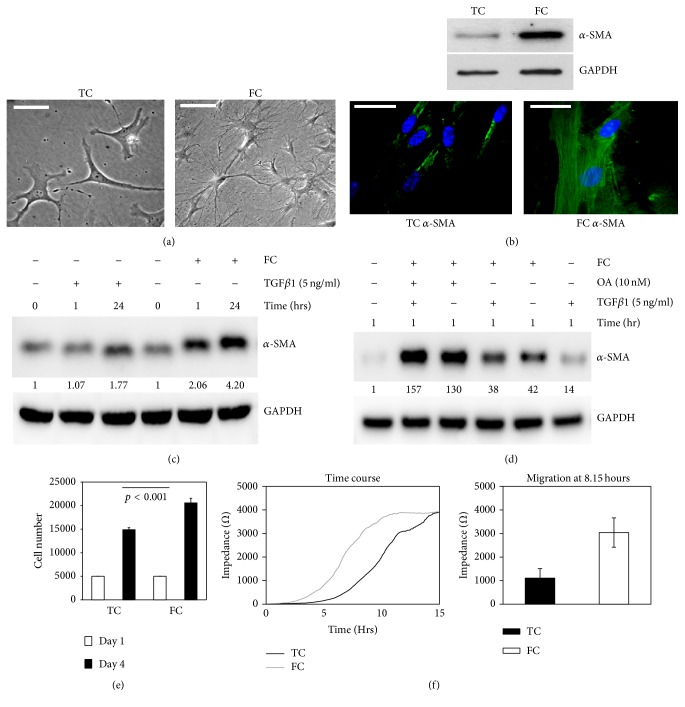
FC promoted the differentiation and proliferation of myofibroblasts. (a) Image of cell morphology on TC (left panel) and FC (right panel); (b) analysis of *α*-SMA expression in fibroblasts by Western blot (top) and immunofluorescence (bottom) at day 4 on TC or on FC. GAPDH was used as a protein loading control. The nucleuses were counterstained with DAPI (blue). ((c) and (d)) *α*-SMA expression in myocardial fibroblasts in response to FC, OA, and TGF *β*1 by Western blots. The number is the ratio of indicated protein/GAPDH according to densitometry assay. (e) The proliferation assay of fibroblasts in TC versus FC (*p* < 0.001). (f) Cell migration (impedance) was measured by ECIS in a time manner (left panel) and its different peaks of TC versus FC (right panel; *p* < 0.005) at 8.15 hrs. Bar = 50 microns.

In a previous study, we found that collagen deposition accumulated in the area of post-MI in mice [15]. To explore the relationship between FC and the differentiation and proliferation of myofibroblasts, the cells were cultured on FC for five days. We observed that more spindle-shaped and reticular shaped fibers appeared in cells on FC compared to the cells on plastic tissue culture dishes (TC) (Figure 1(a)). Meanwhile, *α*-SMA expression was increased in fibroblasts on FC in comparison to those from TC (Figure 1(b)). These results show that FC promoted the differentiation and proliferation of myofibroblasts. To further investigate the differentiation of myofibroblasts regulated by FC compared to other factors, the cells were treated with FC, TGF *β*1, okadaic acid (OA, a PP2A inhibitor), or the combination of these factors. We found that FC could rapidly increase *α*-SMA expression in cardiac fibroblasts (Figure 1(c)) compared to TGF *β*1 treatment. *α*-SMA expression was elevated in the order of FC + OA + TGF*β*1 (157-fold) > FC + OA (130-fold) ≫ FC (42-fold) > FC + TGF *β*1 (38-fold) ≫ TGF *β*1 (14-fold) (Figure 1(d); the numbers are the ratio of *α*-SMA/GAPDH). These findings support that FC strongly enhanced fibroblast differentiation. Low PP2A activation may synergize with FC-induced integrin signaling during this process. TGF *β*1 might play a role at a later time in response to cell differentiation in our case. After the fibroblasts were cultured for 4 days, cell proliferation was increased on FC (increase of 34%) compared to those that grew on TC (Figure 1(e), *p* < 0.001). To ensure that the myofibroblasts were more active on FC, cell migration was measured via ECIS method. The results showed that myofibroblasts on FC had a higher capacity of migration (highest peak at 8.15 hours) than those on TC (highest peak at 15 hours; Figure 1(f)). These results suggest that FC promoted the differentiation and proliferation of myofibroblasts.

### 3.2. FC Induced Low Expressions of *α*2*β*1 Integrin and PTEN

**Figure 2:**
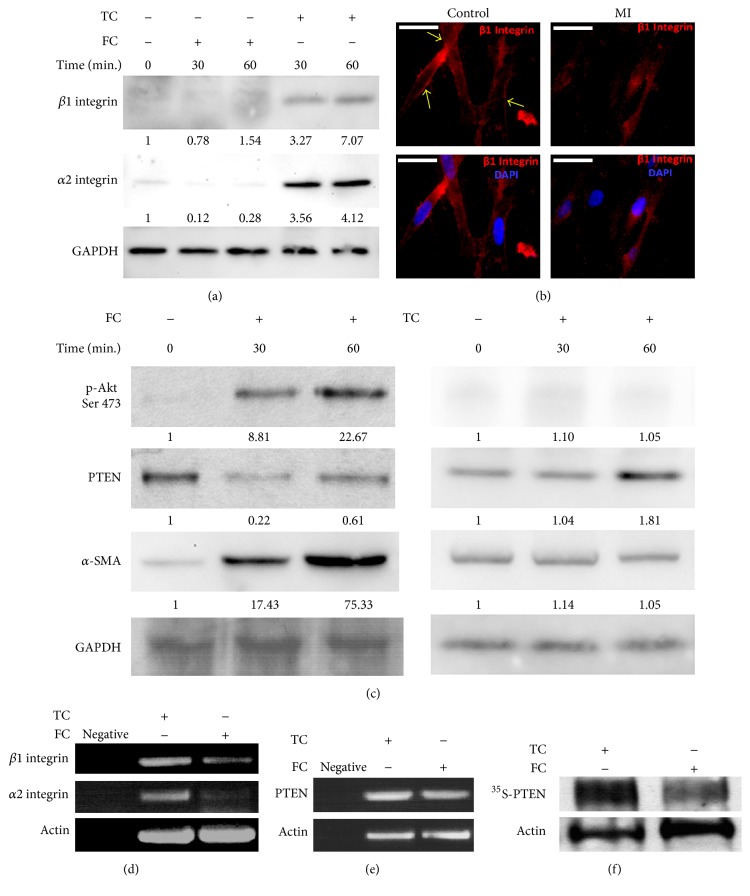
Low expression of *α*2*β*1 integrin and PTEN was respond to FC. (a) Western blots showed that both *α*2 and *β*1 integrin were expressed on TC or FC. (b) *β*1 integrin expression was in fibroblasts of control or the lesion of MI in mice by immunofluorescence. Bar = 50 microns. (c) The expression levels of p-AKT, AKT, PTEN, and *α*-SMA on FC by Western blot on FC and TC. ((d) and (e)) The total RNA of *α*2 and *β*1 integrin, actin, and PTEN was analyzed by semiquantitative RT-PCR. (f) PTEN stability was measured by Pulse-Chase assay on TC or FC. The number is the ratio of indicated protein/GAPDH or AKT according to densitometry assay.

Based on the above data, we then inspected molecules that were involved in mediating the differentiation and proliferation of myofibroblasts on FC. Since *α*2*β*1 integrin is a main receptor of FC, we decided to investigate *α*2*β*1 integrin expression in mouse cardiac fibroblasts. As expected, we found that *α*2*β*1 integrin expression was lower on FC compared to that on TC (Figure 2(a)). Next, we examined *β*1 integrin expression in mouse fibroblasts isolated from lesions of MI or normal tissues by immunofluorescence. We found that *β*1 integrin expression was reduced significantly when compared with control (Figure 2(b)). Meanwhile, PTEN expression was also decreased, and levels of phosphorylated AKT (p-AKT) and *α*-SMA were elevated in response to TC in a time-dependent manner by Western blot (Figure 2(c)). The results were consistent with enhanced cell migration and changes of cellular morphology in response to FC, further supporting that low expression of *α*2*β*1 integrin and PTEN enhanced the differentiation of myofibroblasts. To further explore how *α*2*β*1 integrin and PTEN expressions were regulated, we examined the transcription level of *α*2 and *β*1 integrin and PTEN by semiquantitative RT-PCR. Interestingly, we found that mRNA levels of both *α*2 and *β*1 integrin were decreased (Figure 2(d)), but mRNA of PTEN did not change on FC, compared to the ones on TC (Figure 2(e)). To study the mechanism of PTEN regulation on FC, PTEN stability was further investigated by the Pulse-Chase assay. The result showed that PTEN protein level in the cells was rapidly degraded on FC compared to the one on TC (Figure 2(f)). Together, these results suggest that low *α*2*β*1 integrin was induced on FC, which in turn led to the activation of AKT and an increase in *α*-SMA via PTEN degradation.

### 3.3. FC Enhanced the Differentiation and Proliferation of Myofibroblasts by Lowering *α*2*β*1 Integrin Expression

**Figure 3:**
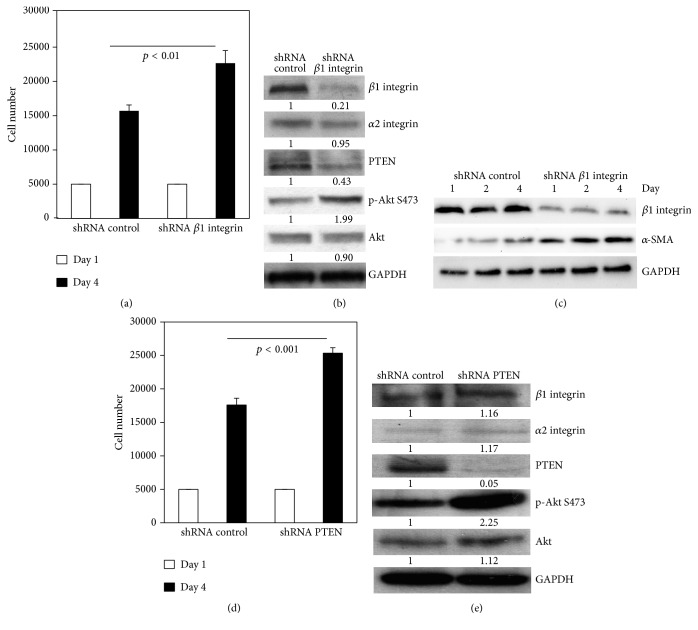
Knocking down *β*1 integrin enhanced the differentiation and proliferation in myofibroblasts on FC. (a) Proliferation assay (*p* < 0.01) and (b) the expression levels of *α*2 and *β*1 integrin, PTEN, p-AKT, and AKT were analyzed in shRNA-control or shRNA-*β*1 integrin transfected fibroblasts on FC at day 4 by Western blot. (c) *α*-SMA expression by Western blots in shRNA-control or shRNA-*β*1 integrin transfected fibroblasts. (d) The proliferation assay (*p* < 0.001). (e) The expression levels of *α*2 and *β*1 integrin, PTEN, p-AKT, and AKT in shRNA-control or shRNA-PTEN transfected fibroblasts on FC at day 4 by Western blot.

To further verify whether low *β*1 integrin expression could regulate the differentiation and proliferation of myofibroblasts on FC, *β*1 integrin was knocked down by shRNA using lentivirus and confirmed by Western blot (Figures 3(b) and 3(c)). We found that knocking down *β*1 integrin increased proliferation of fibroblasts on FC compared to that of the control by Western blot (*p* < 0.01; Figure 3(a)). In addition, the PTEN level decreased. As a result, the p-AKT and *α*-SMA levels were elevated (Figures 3(b) and 3(c)), while *α*2 integrin expression did not change. These results further indicate that low *β*1 integrin expression promoted the differentiation and proliferation of myofibroblasts on FC. We further studied myofibroblast proliferation via gain and loss of function of PTEN. The results showed that knocking down PTEN by shRNA promoted cell proliferation (Figure 3(d); *p* < 0.001). As expected, *α*2 and *β*1 integrin expression were unchanged, but p-AKT was markedly elevated (Figure 3(e)). On the contrary, overexpressing wild type PTEN with adenovirus in cells inhibited cell proliferation (data not shown). Overall, low expression of *β*1 integrin promoted the differentiation and proliferation in myofibroblasts via the interaction of *α*2*β*1 integrin with FC through the PTEN and AKT pathway.

### 3.4. PP2A Activity Was Regulated by *α*2*β*1 Integrin on FC

**Figure 4:**
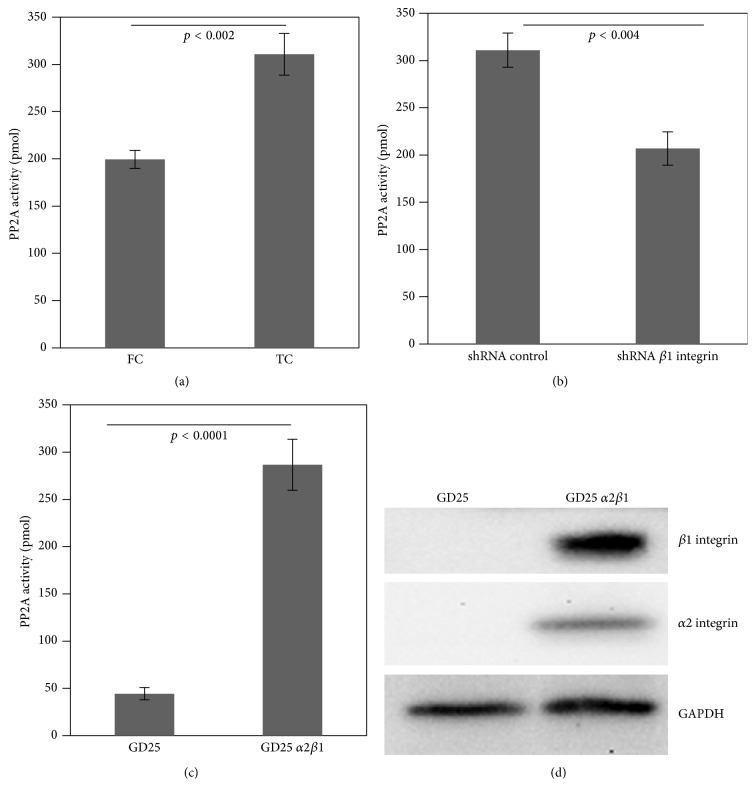
PP2A activity was regulated by *α*2*β*1 integrin on FC. (a) Analysis of PP2A activity in fibroblasts on TC or FC (*p* < 0.002). (b) Analysis of PP2A activity in shRNA-control or shRNA-*β*1 integrin transfected fibroblasts on FC (*p* < 0.004). (c) Analysis of PP2A activity in GD25 *α*2*β*1 integrin-null cells or GD25 reconstituted with *α*2*β*1 integrin on FC (*p* < 0.0001). (d) Western blots showed the protein levels of *α*2 and *β*1 integrin in GD25 without or with *α*2*β*1 integrin on FC.

In accordance with previous reports, *α*2*β*1 integrin was not only a major receptor of FC, but also an activator of PP2A [14]. Based on our data, *α*2*β*1 integrin expression was low in fibroblasts on FC. We investigated whether low *α*2*β*1 integrin resulted in altered PP2A activity in fibroblasts. We first examined PP2A activity in fibroblasts on FC. Although we could not detect the expression changes of PP2Ac in fibroblasts, PP2A activity was significantly decreased on FC, compared to that on TC (Figure 4(a); *p* < 0.002). To further confirm whether *β*1 integrin expression was correlated with PP2A activity, we knocked down *β*1 integrin by shRNA in fibroblasts. We found that PP2A activity was markedly reduced in fibroblasts with *β*1 integrin shRNA on FC, compared to control-shRNA (Figure 4(b), *p* < 0.004). Furthermore, we wanted to confirm whether PP2A activity was related to low *α*2*β*1 integrin. We examined PP2A activity in both GD25 *α*2*β*1 integrin-null fibroblasts and GD25 fibroblasts reconstructed with *α*2*β*1 integrin cultured on FC. Notably, GD25 cells could not attach well on FC (Figure 4). Similar to the results of knock-down *β*1 integrin, GD25 fibroblasts had very low PP2A activity. In contrast, GD25 fibroblasts with reconstituted *α*2*β*1 integrin had higher PP2A activity on FC (5-fold; Figure 4(c)).

### 3.5. PP2A Regulated the Differentiation and Proliferation of Myofibroblasts on FC

**Figure 5:**
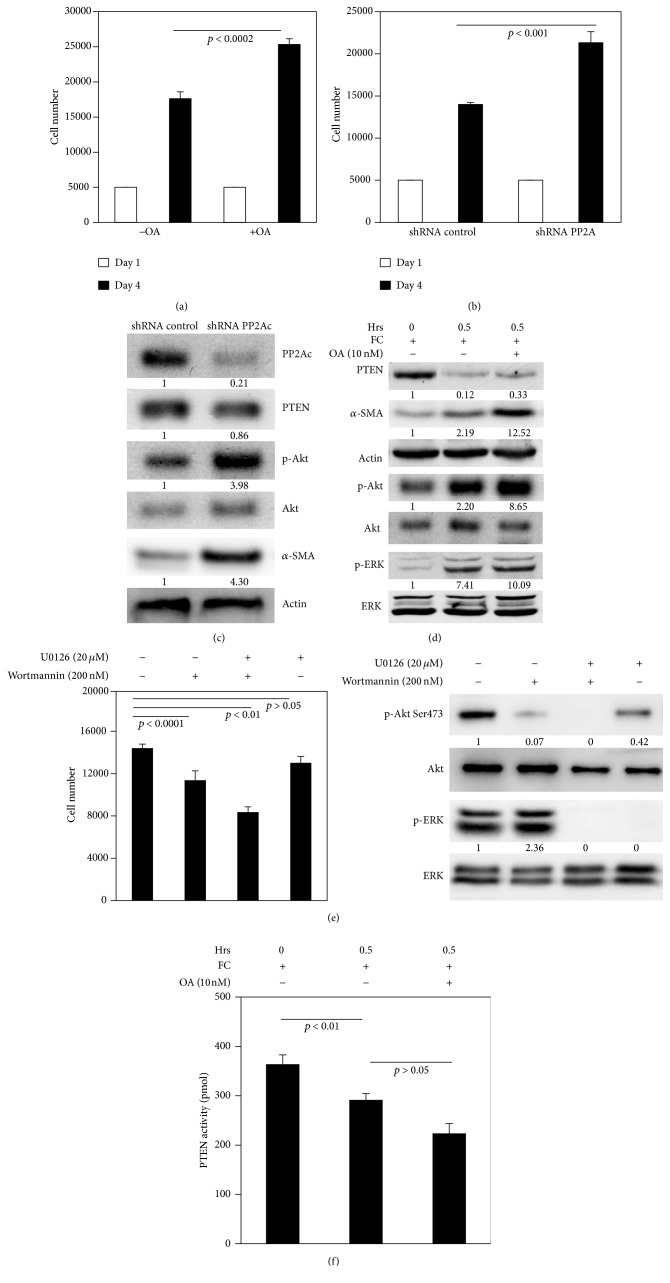
Low PP2A activity promoted the differentiation and proliferation in cardiac fibroblasts on FC. (a) The proliferation assay of fibroblasts with or without okadaic acid treatment on FC (*p* < 0.0002). (b) The proliferation assay of fibroblasts (*p* < 0.001). (c) Western blots showed the expressions of PP2Ac, PTEN, AKT, p-AKT, *α*-SMA, and actin at day 4 in shRNA-control or shRNA-*β*1 integrin transfected fibroblasts on FC. (d) The expressions of PTEN, *α*-SMA, actin, p-AKT, AKT, p-ERK, and ERK by Western blot in fibroblasts with or without okadaic acid treatment on FC. (e) Analysis of proliferation (left panel) and of the expressions of p-AKT, AKT, p-ERK, and ERK by Western blot in fibroblasts treated with control, UO126, wortmannin, or UO126 + wortmannin on FC at day 4 (right panel). (f) Analysis of PTEN activity in fibroblasts treated with or without okadaic acid treatment on FC. Representative immunoblots from three independent experiments are shown.

Since low PP2A activity was correlated with low *α*2*β*1 integrin expression, we wanted to address the question whether low PP2A activity regulated proliferation of myofibroblasts. The fibroblasts were treated by PP2A inhibitor OA (10 nM) for 4 days, or PP2A was knocked down by shRNA. First, we found that proliferation was increased by both OA treatment and PP2Ac knock-down (Figures 5(a) and 5(b)). Secondly, PTEN expression was only changed mildly when PP2Ac expression was knocked down by 79% (Figure 5(c)). Interestingly, p-AKT level was markedly elevated (3.98-fold) and *α*-SMA expression was also increased in the cells with shRNA-PP2Ac, compared to the cells with control-shRNA (Figure 5(c)). This result indicated that PP2A mainly targeted AKT, rather than PTEN. Furthermore, we examined how FC regulated the differentiation and proliferation of myofibroblasts via PP2A. The cells were cultured on FC without or with OA as a function of time. Again, we found that PTEN expression was reduced on FC. But PTEN expression had a little change, compared to the cells with or without OA. However, the expressions of p-AKT and *α*-SMA were significantly increased (about 3.93-fold or 5.7-fold, resp., Figure 5(d)). We also observed that phosphorylated ERK in fibroblasts was increased on FC, but phosphorylated ERK did not change when treated with OA. This finding further supported that AKT was a major target of PP2A, which was involved in regulation of the differentiation of myofibroblasts.

To further investigate how AKT or ERK signaling contributed to the regulation of fibroblast proliferation, the fibroblasts were treated with wortmannin (an AKT inhibitor) or U0126 (an ERK inhibitor) or both for 4 days. Fibroblasts not treated with those agents served as control. The results showed that inhibition of ERK mildly suppressed cell proliferation (*p* > 0.05; Figure 5(e)), but inhibition of AKT significantly suppressed cell proliferation (*p* < 0.01; Figure 5(e)). However, inhibition of both AKT and ERK remarkably suppressed cell proliferation (*p* < 0.001; Figure 5(e)), which indicated that ERK alone might have a minor role in regulating the differentiation and proliferation of myofibroblast. In our case, PTEN/AKT signal might be more effective in terms of affecting differentiation and proliferation. PTEN activity in the cells on FC was inhibited more than that in the control (down 34%; Figure 5(f)), but PTEN activity in the cells showed no difference between the cells without or with OA treatment (*p* > 0.05), indicating PP2A may only have minor effect on PTEN. Taken together, these results showed that integrin regulated the differentiation and proliferation of cardiac myofibroblasts through *α*2*β*1 integrin/PTEN/PP2A signaling.

## 4. Discussion

Cardiac fibrosis is a key contributor to heart failure in post-MI patients, but the molecular mechanisms underlying their fibrogenicity remain undefined. In this study, we mimicked the 3-dimensional ECM of post-MI cells in vivo via FC matrices. Our data revealed that low levels of *α*2*β*1 integrin and its interaction with FC were closely related to inappropriately low PTEN and PP2A activity, leading to abnormal activation of AKT, and the differentiation and proliferation of myofibroblasts.

Based on our results, the cardiac fibroblasts were rapidly differentiated to myofibroblasts in response to FC induction. FC enhanced the migratory and proliferative capability of myofibroblasts, which was consistent with the morphological change and high levels of *α*-SMA expression (Figures 1(a)–1(e)). In this study, we found that low expression of *α*2*β*1 integrin in response to FC contributed to the increased AKT activity and *α*-SMA expression in cardiac fibroblasts. The increased AKT activation as a result of decreasing PTEN activation is required in the processes of differentiation and proliferation of myofibroblast in response to FC. Our data showed that FC strongly enhanced the differentiation of myocardial fibroblasts to myofibroblasts. The cumulative evidence suggested that TGF *β*1 was linked to the differentiation of fibroblast to myofibroblast when fibrosis developed [23–27]. However, during myofibroblastic transition, TGF *β*1 was also secreted, which enabled fibroblast differentiation via integrin and collagen [28, 29]. Whether TGF *β*1 synergizes with integrin to enhance cardiac myofibroblast differentiation requires further investigation.

Furthermore, we demonstrated that *α*2*β*1 integrin expression was decreased in myocardial fibroblasts on FC at both the protein and mRNA level. However, decreased PTEN activity was caused by the degradation of PTEN protein rather than upregulation at the transcription level. By knocking down *β*1 integrin in fibroblasts and analyzing GD25 *β*1 integrin-null cells and the GD25 cells reconstituted with *α*2*β*1 integrin, we further confirmed that *α*2*β*1 integrin is an important regulator in mediating the differentiation and proliferation of myofibroblasts via decreasing PTEN activity and increasing AKT activity in response to FC.

The phosphorylation and dephosphorylation of proteins mediated by PP2A play an important role in the control of a variety of cellular processes. PP2A has many targets, such as AKT, ERK, and histone deacetylases [17, 24]. We found that FC reduced PP2A activity in fibroblasts, which was correlated with low a2*β*1 integrin expression, consistent with the previous studies [14, 17]. To better understand what the main targets of PP2A were in myofibroblasts in cardiac fibrosis, we detected phosphorylated AKT and found it was drastically elevated (increased by up to 65%) due to inhibition by PP2A. However, PP2A inhibitor did not affect the level of expression of phosphorylated ERK (Figure 5(d)). PTEN expression and activity were mildly affected, suggesting that PP2A might mainly target AKT. In addition, *α*-SMA expression was markedly elevated by PP2A inhibition and knock-down. These results indicated that suppression of PP2A might impact myofibroblasts differentiation via enhancement of AKT activity (Figures 5(b) and 5(d)). In this case, PTEN might not be a major target of PP2A because PTEN activity was not significantly affected by treatment with the PP2A inhibitor (Figure 5(d)). Based on our results, FC increased ERK activity, which has been reported to cause cardiac fibrosis [30, 31]. However, our data did not show that ERK inhibition significantly changed the fibroblast proliferation (Figure 5(e)). However, cell proliferation was drastically suppressed by the combination of wortmannin and UO126. These results implied that ERK might synergize with PTEN and AKT signaling to regulate cell proliferation.

In this study, we demonstrated that FC induced the differentiation and proliferation of myofibroblasts in cardiac fibrosis via pathologically low *α*2*β*1 integrin expression. Abnormal activation of AKT was due to the inappropriately low activity of PTEN and PP2A. The integrin, PTEN, and PP2A pathways may provide a potential therapeutic target for cardiac fibrosis.
